# Effect of traditional Chinese exercise on older patients with diabetes mellitus: a systematic review and meta-analysis of randomized controlled trials

**DOI:** 10.3389/fendo.2025.1499051

**Published:** 2025-05-08

**Authors:** Weimin Liu, Chong Chin Che, Ping Lei Chui, Zifeng Ma, Jing Chen

**Affiliations:** ^1^ Department of Oncology, Beijing Shijitan Hospital, Capital Medical University, Beijing, China; ^2^ Department of Nursing Science, Faculty of Medicine, Universiti Malaya, Kuala Lumpur, Federal Territory of Kuala Lumpur, Malaysia; ^3^ Department of Emergency, Beijing Tongren Hospital, Capital Medical University, Beijing, China; ^4^ Party Committee Office, Beijing Shijitan Hospital, Capital Medical University, Beijing, China

**Keywords:** older patient, diabetes mellitus, blood glucose, traditional Chinese exercise, systematic review, meta-analysis

## Abstract

**Objective:**

This systematic review and meta-analysis aimed to evaluate the effects of traditional Chinese exercises (TCEs) on blood glucose, glycosylated hemoglobin (HbA1c), body mass index (BMI), and health-related quality of life in older patients with diabetes mellitus (DM).

**Methods:**

Database searches were systematically conducted across multiple platforms. The review adhered to PRISMA guidelines, utilizing the Cochrane Risk of Bias Assessment Tool to gauge literature quality. Review Manager 5.3 was employed for data evaluation, calculating mean differences to ascertain pooled effect sizes.

**Results:**

This study encompassed 11 randomized controlled trials involving 944 individuals. The results showed that TCEs reduced fasting blood glucose (-0.76, 95% CI [-1.14, -0.38], P = 0.0001), HbA1c (-2.64, 95% CI [-4.81, -0.47], P = 0.02), and BMI (-0.83, 95% CI [-1.42, -0.24], P = 0.006), and improved health-related quality of life. Among the various forms of TCEs, Baduanjin (BDJ) appeared particularly beneficial.

**Conclusions:**

Traditional Chinese exercises can improve blood glucose levels, BMI and quality of life-related indicators to varying degrees in older diabetes patients, and may be a useful complementary therapy for this population.

## 1 Introduction

Diabetes mellitus (DM) encompasses a cluster of metabolic disorders distinguished by elevated blood glucose levels resulting from multiple etiologies. In the last 40 years, a series of lifestyle, technology, and social development changes led to the global rise of diabetes mellitus (1, 2). As of 2021, the global population of individuals with diabetes mellitus reached 537 million (3). China accounted for 116 million diabetes mellitus patients, which makes it the country with the highest number of people affected by diabetes mellitus globally (4). However, this absolute figure reflects its large opulation base. In terms of relative prevalence, Western countries—particularly those with higher obesity rates—bear a proportionally greater burden of diabetes. With an increasingly ageing population, increasing morbidity of diabetes mellitus, and increasing life expectancy, older patients with DM have emerged as the dominant population with diabetes (5, 6). Diabetes mellitus will produce serious short- and long-term medical complications without appropriate treatment or management and impose a considerable economic burden on the healthcare system.

An increasing body of evidence has substantiated the significant physiological and health advantages associated with exercise (7–10), demonstrating its preventive or delaying effects on the onset of diabetes. Thus, regular exercise is advised for all patients with diabetes mellitus as a component of their blood glucose regulation and health management. Older individuals with diabetes mellitus commonly exhibit a range of concurrent chronic conditions, including osteoarthropathy, which impairs their ability to walk, peripheral neuropathy and severe myopathy which may increase their susceptibility to falls and result in reduced balance ability (11, 12). Older patients with diabetes mellitus should be encouraged to engage in regular physical activity with low to moderate intensity. The recommended physical activity should be individualized, easy to perform, and sustainable. It should also include exercises that promote muscle-building.

Moreover, older patients should be free to choose their preferred exercise mode and duration (30 to 45 min/d) (13, 14). Traditional Chinese exercises (TCEs) are traditional Chinese fitness modalities that combine both internal and external training, rigidity, and flexibility, which mainly include Baduanjin (BDJ), Tai Chi (TC), Wu Qin Xi (WQX), Yi Jin Jing (YJJ), and Qi Gong (QG), etc. As a low-intensity exercise with Chinese characteristics, traditional Chinese exercises have become an active health management modality for patients with DM. This is attributed to its inherent advantages of being safe, economical, and not restricted by space and time (15, 16). Compared with conventional aerobic or resistance exercise, traditional Chinese exercises are easier, and the amount and intensity of exercise can be appropriately adjusted according to individual fitness (17), which may be more in line with the exercise needs of older populations.

Despite the positive effects of traditional Chinese exercises on the health management of patients with diabetes mellitus, weaknesses in study design and methodology have been identified in this research. Currently, most of the research in this field utilizes randomized controlled trials (RCTs) to validate the clinical effectiveness of individual traditional exercise therapy for diabetes mellitus. Previous systematic evaluations focus on the health benefits of traditional Chinese exercises for adults with DM, which may not reflect the clinical efficacy of traditional Chinese exercises for older individuals with diabetes mellitus. Therefore, five traditional Chinese exercises commonly employed in clinical practice were included in this study to address these issues. A systematic review and meta-analysis were employed to integrate relevant clinical evidence. A quantitative statistical analysis was conducted to comparatively assess the clinical effectiveness of five distinct traditional Chinese exercises therapies (Baduanjin, Tai Chi, Wu Qin Xi, Yi Jin Jing, Qi Gong) in the treatment of older individuals with diabetes mellitus. The aim of this systematic review and meta-analysis was to serve as a reference for selecting exercise intervention therapies that demonstrate greater effectiveness in the clinical treatment of older individuals with diabetes mellitus.

## 2 Methodology

### 2.1 Protocol and registration

This review was carried out as per the Preferred Reporting Items for Systematic Evaluation and Meta-Analysis (PRISMA) guidelines (18). The protocol was registered to the PROSPERO database (No. CRD42023413734).

### 2.2 Search strategy

A comprehensive search on the website for five RCTs related to the use of TCEs in the treatment of DM. Search criteria are limited to publications from the database inception up to March 16, 2023 and included only English or Chinese language studies. Databases searched included PubMed, Web of Science, Embase, Cochrane Library, China National Knowledge Infrastructure, and Wanfang Data Knowledge Service Platform. The MeSH and free-word terms were combined to determine peer-reviewed articles from TCEs-based RCTs involving older patients with DM. Search terms related to type 2 diabetes mellitus, type 1 diabetes mellitus, TCEs, Tai Chi, BaduanJin, Yi Jin Jing, Qigong, and Wu Qin Xi were employed. The detailed search strategy is listed in the appendix. Additionally, pertinent references were tracked in the literature, and communication was established with the corresponding authors when a complete report was unavailable, or the document lacked essential data. The search is guaranteed to be comprehensive, thereby eliminating the possibility of any valuable research data being overlooked.

### 2.3 Inclusion and exclusion criteria

Two reviewers identified the literature independently (WM L, ZF M), and any disagreements were addressed through discussion or by seeking input from a third party for resolution. The studies included in this analysis satisfied the following criteria: (1) The study was designed as a RCT. (2) The study population was older adults (≥ 60 years) with DM, medically diagnosed with a fasting blood glucose (FPG/FBG) level of 7.0 mmol/L (126 mg/dL) and above, a 2-hour glucose of 11.1 mmol/L (200 mg/dL) or more, or a glycosylated hemoglobin (HbA1c) of 6.5% (19, 20) and above. (3) Interventions: the control group was conventional care or standard diabetes treatment, with or without any type of other routine exercises (jogging and swimming, etc.), and the treatment group was treated with one of the five traditional exercise therapies (BDJ, TC, WQX, YJJ, QG) under the same standard diabetes treatment (4) Study results must include FPG/FBG, HbA1c, 2 hours postprandial blood glucose (2hPG/PPG), body mass index (BMI), 36-itemShort-Form (SF-36), and DM-specific quality of life scale (DSQoL). (4) Exclusion criteria: studies using the voluntary grouping principle and other non-RCTs, case reports, reviews, duplicate publications, empirical summaries, studies with incomplete outcome indicators for which valid data could not be extracted, studies with inconsistent interventions, and conference papers.

### 2.4 Data extraction and synthesis

The extraction of data in the study was conducted independently by two researchers (WM L and ZF M). A data extraction form was prepared according to the CONSORT statement (21) with the following content: main author, time of publication, article source, country of publication, diagnostic criteria, study design, study population, sample size, baseline, and endpoint values (mean and standard deviation), trial duration, and details of the intervention. During data extraction, any conflicts or uncertainties encountered in the results were addressed by engaging in discussions with a third reviewer and reaching a consensus to resolve them.

### 2.5 Outcome measures

The primary outcome indicators of the research included (1) FPG/FBG; (2) HbA1c. Secondary outcome indicators included (1) 2hPG/PPG; (2) BMI; (3) SF-36; (4) diabetes-specific quality of life scale (DSQoL).

### 2.6 Risk of bias assessment

The Cochrane Risk of Bias Assessment Tool (22) was utilized to evaluate the quality of the literature. The main areas included the following: Selection bias, such as random sequence generation and allocation concealment; implementation bias, including blinding of investigators and subjects; measurement bias, including blinded evaluation of study outcomes; follow-up bias, including completeness of outcome data; reporting bias, or selective reporting; and other sources of bias. Two investigators independently performed assessments, and any disagreements were subject to discussion for resolution. Alternatively, a third party could be invited to provide consultation and negotiate for decision.

### 2.7 Statistical analysis

Review Manager 5.3 (RevMan 5.3, The Nordic Cochrane Centre, The Cochrane Collaboration, Copenhagen, Denmark) was utilized to analyze the data. As the outcome indicators included in the literature were continuous variables with consistent units of measurement, mean difference (MD) was more suitable for meta-analysis, and 95% confidence intervals (CI) were calculated. In this study, heterogeneity between studies was detected by calculating *I^2^* values. When *I^2^ =* 0, there was no heterogeneity; when 0 < *I^2^* < 50%, heterogeneity was small, and fixed-effect models were used for analysis. At *I^2^* ≥50%, there was a large degree of heterogeneity, and consequently, a random effects model was employed for the analysis. Subgroup analyses were conducted to explore the sources of heterogeneity, specifically focusing on TCEs and intervention duration the heterogeneity is large and the random effects model is used for analysis. We also performed subgroup analyses of TCEs and subgroup analyses of intervention duration to explore the sources of heterogeneity. As recommended by the Cochrane Handbook, potential publication bias was analyzed using RevMan software when the number of included studies exceeded 10. If the plot was symmetrical with an inverted funnel shape, it indicated a relatively low likelihood of publication bias. If the funnel plot was asymmetrical or incomplete, it suggested a higher likelihood of publication bias.

## 3 Results

### 3.1 Search results

**Figure 1:**
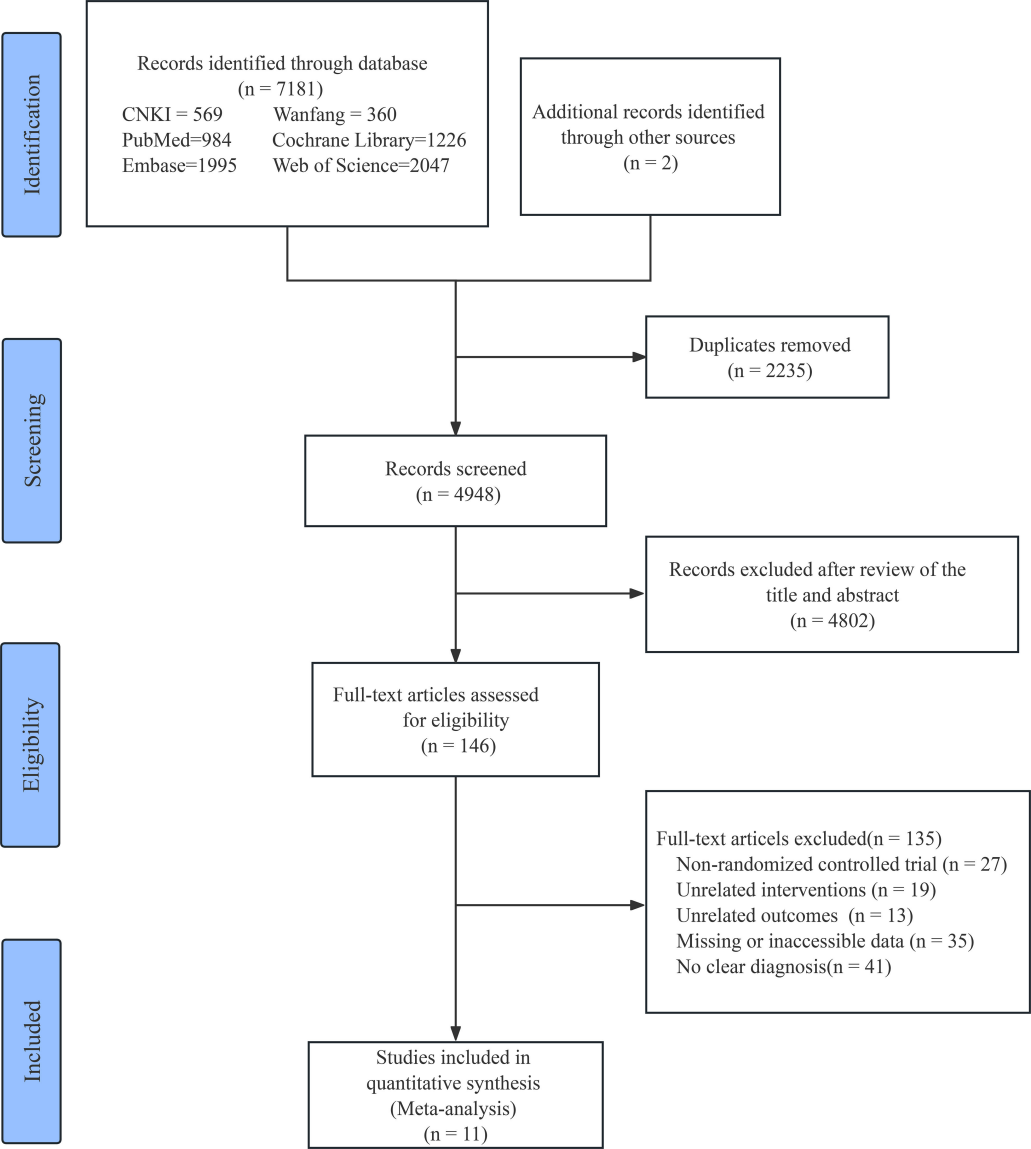
Flow chart of study selection. CNKI, China National Knowledge Infrastructure; Wanfang, Wanfang Data Knowledge Service Platform.

The literature selection, inclusion, and exclusion process is illustrated in **Figure 1**. Overall, 7183 potentially eligible studies were obtained through database and manual searches. After thoroughly assessing the titles and abstracts, 2235 duplicate studies and 4802 irrelevant studies were removed, with 146 studies being left for assessment. These were then fully analyzed and discussed. An additional 135 studies were excluded, of which 27 were non-RCTs, 19 had inconsistent interventions, 13 had unrelated outcomes, 35 had missing or unextractable data, and 41 had no clear diagnostic criteria. The final 11 studies were eligible for inclusion and were analyzed comprehensively.

### 3.2 Study features

All eligible studies included in the review were published within the timeframe of 2003 and 2022. A total of 11 eligible trials (23–33) with 944 participants were utilized in a subsequent meta-analysis. Intervention periods ranged from 3 to 6 months. Intervention frequency ranged from 2 to 7 days per week, and intervention duration ranged from 30 to 60 min per session. Exercise forms included TC in 7 cases, BDJ in 3 cases, and YJJ in 1 case. Nine studies (23–31) reported FPG/FBG, eight studies (23, 26–31, 33) reported HbA1c, and the remaining studies reported 2hPG/PPG, BMI, SF-36, and DSQoL. The basic features of the included studies are illustrated in **Table 1**.

**Table 1:** Basic characteristics of literature included in meta-analysis.

Article, year	Sample size (E/C)	Age (years)		Experimental group			Control group Content	Outcome indicator
		E^a^	C^b^	Content	Frequency (per week, min/times)	Duration (per week)		
Zhou et al., 2011 (23)	63/63	67. 4 ± 9. 23	68. 13 ± 10. 64	BDJ^c^	30min/d, 2d/w	12	Routine exercise	①②⑤⑥
Wang et al., 2003 (24)	10/6	60-70	60-70	TC^d^	60min/d, 7d/w	12	Routine exercise	①⑥
Shi, 2020 (25)	30/30	68.21 ± 4.21	68.59 ± 4.65	BDJ	30min/d, 5d/w	12	Routine exercise	①⑤⑥
Meng, 2014 (26)	100/100	68. 4 ± 3. 2	68. 4 ± 3. 2	TC	Unclear	12	Routine exercise	①②③⑤
Luo, 2021 (27)	40/40	66. 54 ± 10. 32	67. 32 ± 9. 46	BDJ	40min/d, 3d/w	12	regular treatment	①②⑤
Lu et al., 2022 (28)	60/60	66.5 ± 3.68	67.17 ± 2.86	YJJ^e^	30-40min/d, 3d/w	12	regular treatment	①②
Li et al., 2015 (29)	50/50	62.91 ± 2.48	63.27 ± 2.86	TC	40-50min/d, 7d/w	24	Routine exercise	①②⑥
Shen et al., 2019 (30)	52/49	67.8 ± 5.1	66.2 ± 4.6	TC	60min/d, 3d/w	12	Routine exercise	①②④
Cai, 2018 (31)	27/23	64.54	64.51	TC	30min/d, 3d/w	12	No intervention	①②⑥
Tracey Tsang, 2007 (32)	18/20	66 ± 8	65 ± 8	TC	60min/d, 2d/w	16	sham-exercise	③
Paul Lam, 2008 (33)	28/25	63.2 (8.6)	60.7 (12.2)	TC	60min/d, 2d/w	12	Routine exercise	②③

a E, Experimental group.

b C, Control group.

c BDJ, Baduanjin.

d TC, Tai Ji.

e YJJ, Yijinjing; ①=FPG/FBG; ②=HbA1c; ③=SF-36; ④=DSQoL; ⑤=2hPG/PPG; ⑥=BMI.

### 3.3 Risk of bias

**Figure 2:**
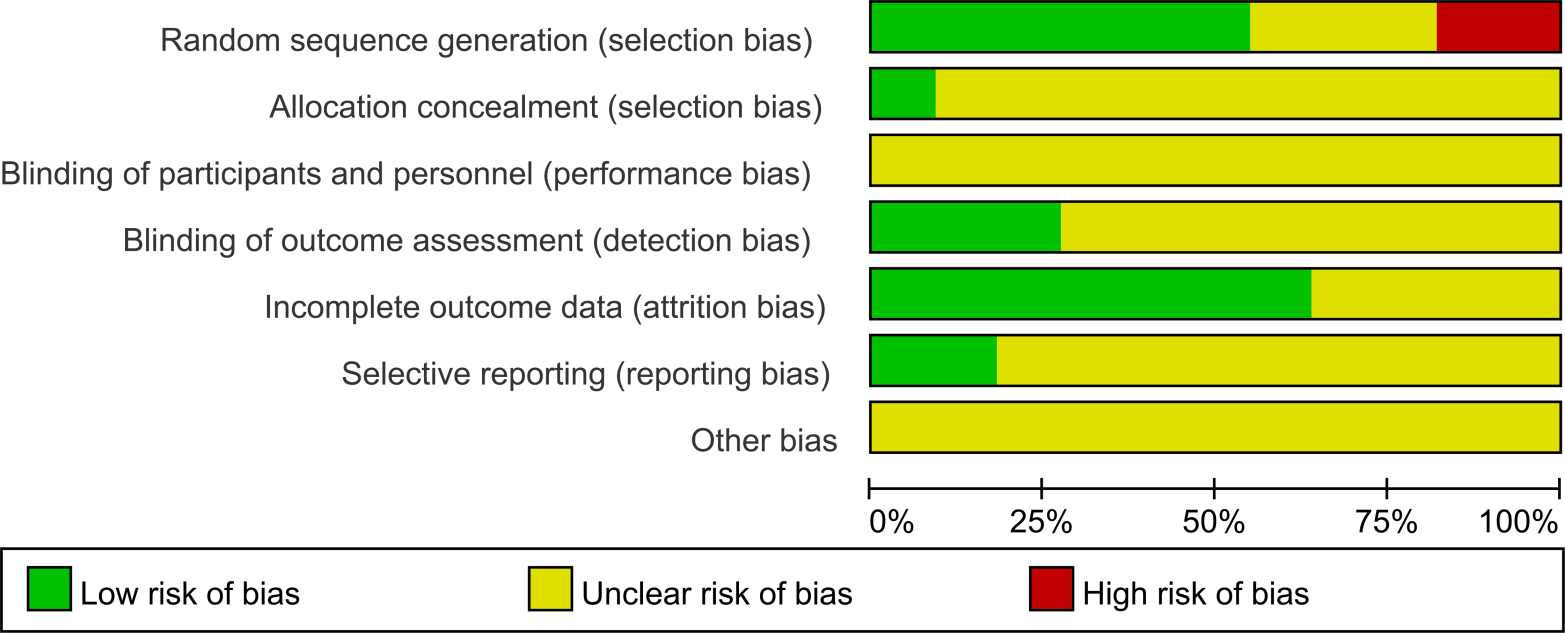
Risk of bias assessment.

**Figure 3:**
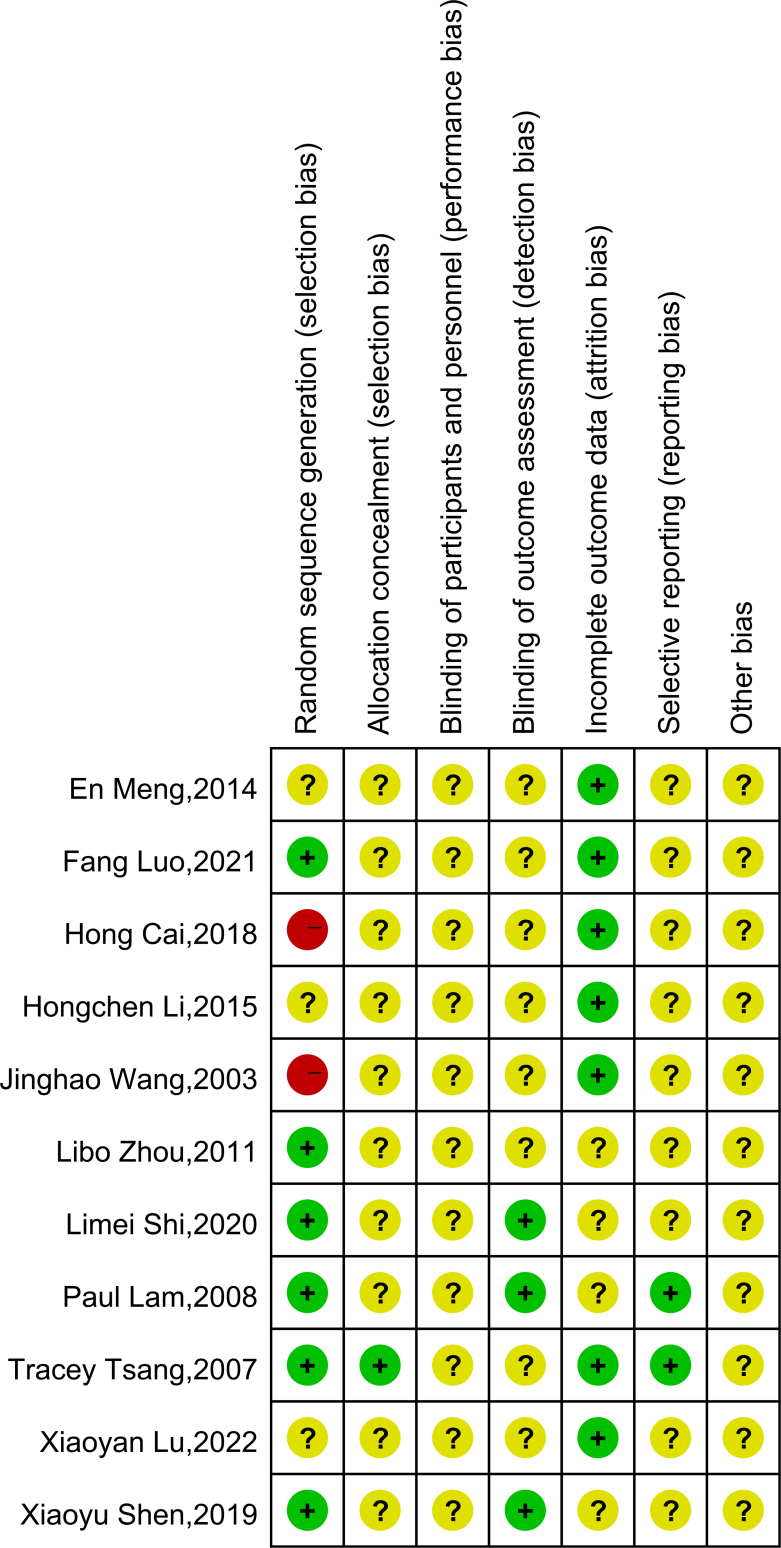
Summary of risks of bias.

The risk of bias assessment is illustrated in **Figure 2**. The summary of the risk of bias is depicted in **Figure 3**. Only 6 of all included studies described the randomization method in detail. Four (23, 25, 27, 30) used randomized number tables, and two studies (32, 33) used number tables generated by computerized randomization procedures. Only one of the 11 studies (32) described specific allocation concealment. TCEs interventions are nearly impossible to conduct using the blind method. Three studies (25, 30, 33) described control of measurement bias by normative measurement with a professional assessment. Seven studies (24, 26–29, 31, 32) described specific follow-ups with complete outcome data. Two studies (32, 33) reported prespecified outcomes with a low risk of bias in their methods section. Finally, because some of the information is not described in detail in all the studies, it was determined that the risk of other sources of bias was unknown.

### 3.4 Effects of TCEs on primary outcomes

#### 3.4.1 FPG/FBG

**Figure 4:**
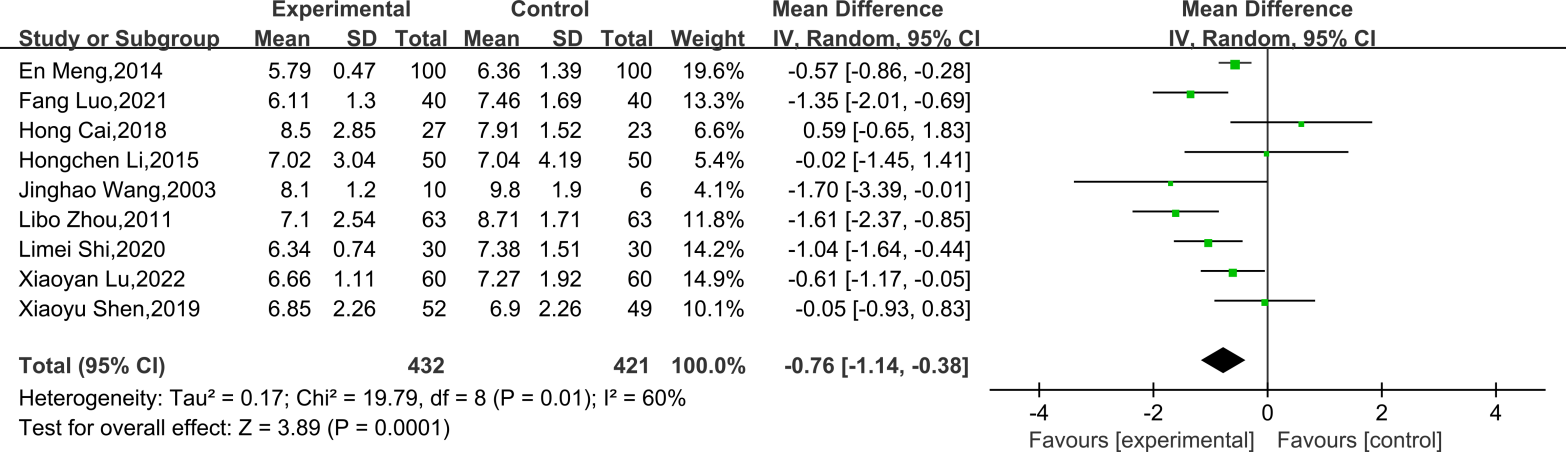
Forest plot of meta-analysis of FPG/FBG effect size.

**Figure 5:**
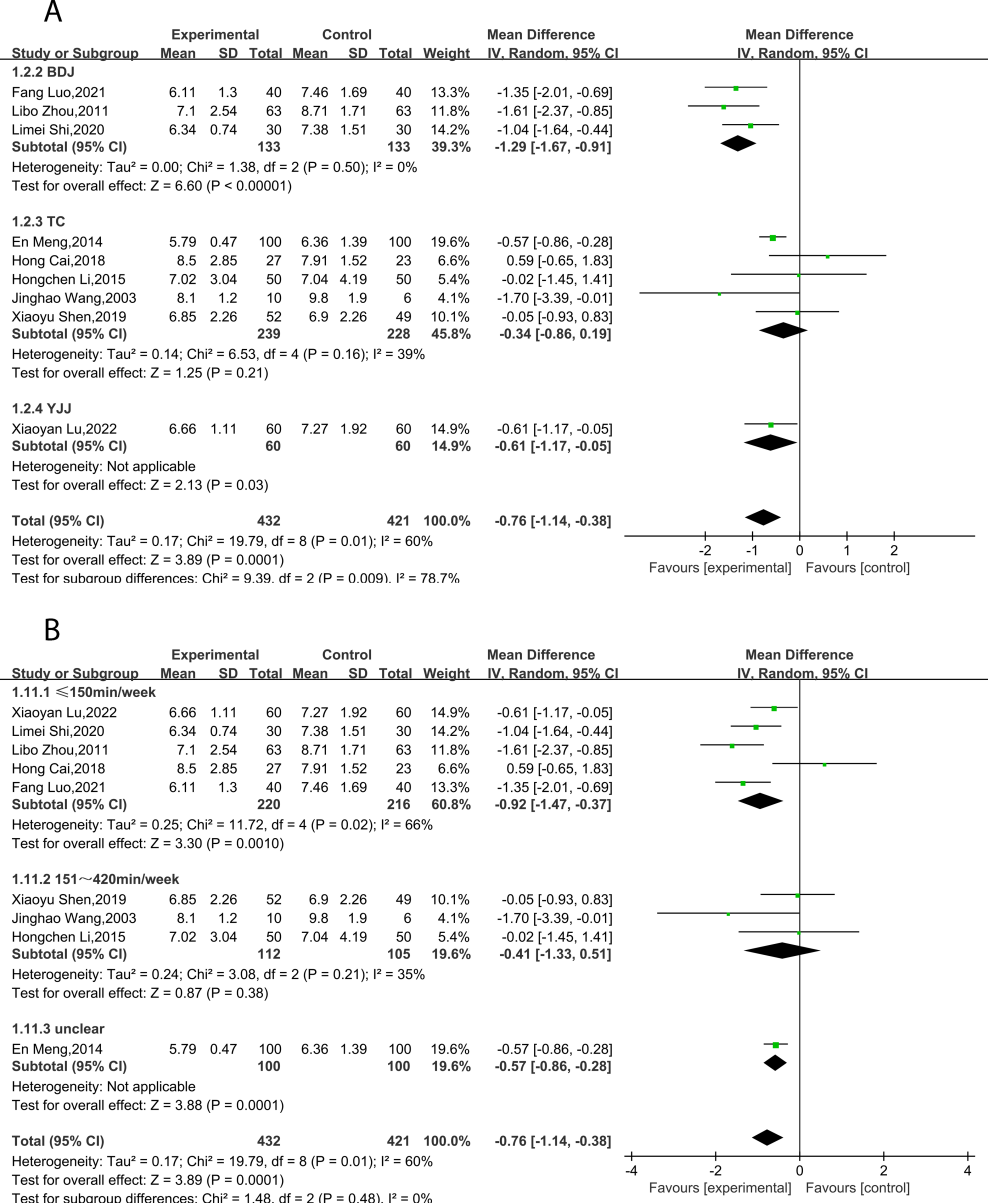
Forest plot of meta-analysis of FPG/FBG effect size. **(A)** Effects of different types of TCEs on FPG/FBG. **(B)** Effects of TCEs on FPG/FBG in different treatment courses.

Nine studies (23–31) reported FPG/FBG, including 853 subjects. All studies tested subjects at baseline before the trial, and the differences were not statistically significant. Heterogeneity is large (*P* = 0.01, *I²* = 60%), therefore, a random effects model was utilized for analysis. The combined results demonstrated that TCEs considerably decreased FPG/FBG (MD = -0.76, 95% CI [-1.14, -0.38], *P* = 0.0001; **Table 2**, **Figure 4**). Subgroup analysis indicated that the intervention effect was greater for the BDJ than for the other TCEs (MD = -1.29, 95% CI [-1.67, -0.91], P < 0.00001; **Table 2**, **Figure 5A**). Subgroup analysis of intervention duration demonstrated that the greatest intervention effect could be achieved when TCEs ≤150 min/week (MD = -0.92, 95% CI [-1.47, -0.37], P = 0.0010; **Table 2**, **Figure 5B**).

**Table 2:** Summary of the meta-analysis results of included studies.

Outcome	Number of RCTs	No. of participants	MD(*95%CI*)	Heterogeneity	*I^2^(%)*	*p*
Main outcomes						
FPG/FBG	9	853	-0.76 [-1.14, -0.38]	0.01	60	0.0001
Different types of TCEs						
BDJ	3	266	-1.29 [-1.67, -0.91]	0.5	0	< 0.00001
TC	5	467	-0.34 [-0.86, 0.19]	0.16	39	0.21
YJJ	1	120	-0.61 [-1.17, -0.05]	N/A^a^	N/A	0.03
Intervention duration/week (min/week)						
≤150	5	436	-0.92 [-1.47, -0.37]	0.02	66	0.001
151∼420	3	217	-0.41 [-1.33, 0.51]	0.21	35	0.38
unclear	1	200	-0.57 [-0.86, -0.28]	N/A	N/A	0.0001
HbA1c	8	830	-2.64 [-4.81, -0.47]	< 0.00001	99	0.02
Different types of TCEs						
BDJ	2	206	-0.76 [-1.16, -0.37]	0.49	0	0.0002
TC	5	504	-3.76 [-7.70, 0.19]	< 0.00001	100	0.06
YJJ	1	120	-0.79 [-1.25, -0.33]	N/A	N/A	0.0008
Intervention duration/week (min/week)						
≤150	5	429	-3.95 [-8.47, 0.57]	< 0.00001	100	0.09
151∼350	2	201	-0.49 [-2.66, 1.69]	< 0.00001	96	0.66
unclear	1	200	-0.54 [-0.71, -0.37]	N/A	N/A	< 0.00001
Secondary outcomes						
2hPG/PPG	4	466	-1.19 [-1.89, -0.49]	0.01	72	0.0008
Intervention duration/week (min/week)						
≤150	3	266	-0.94 [-1.82, -0.06]	0.06	65	0.04
unclear	1	200	-1.71 [-2.13, -1.29]	N/A	N/A	< 0.00001
BMI	5	352	-0.83 [-1.42, -0.24]	0.38	5	0.006
Intervention duration/week (min/week)						
≤150	3	236	-0.76 [-1.42, -0.11]	0.24	29	0.02
151∼420	2	116	-1.10 [-2.46, 0.25]	0.27	16	0.11
SF-36	3	291	4.95 [1.03, 8.87]	0.93	0	0.01
DSQoL	1	101	-5.60 [-9.84, -1.36]	N/A	N/A	0.010

a N/A, Not applicable.

#### 3.4.2 HbA1c

**Figure 6:**
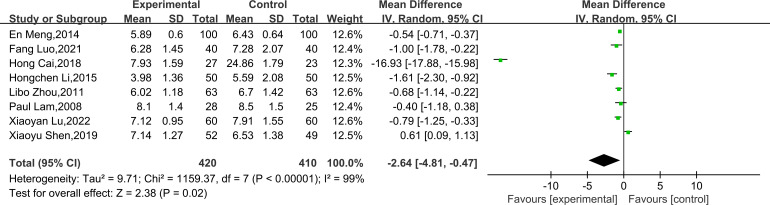
Forest plot of meta-analysis of HbA1c effect size.

**Figure 7:**
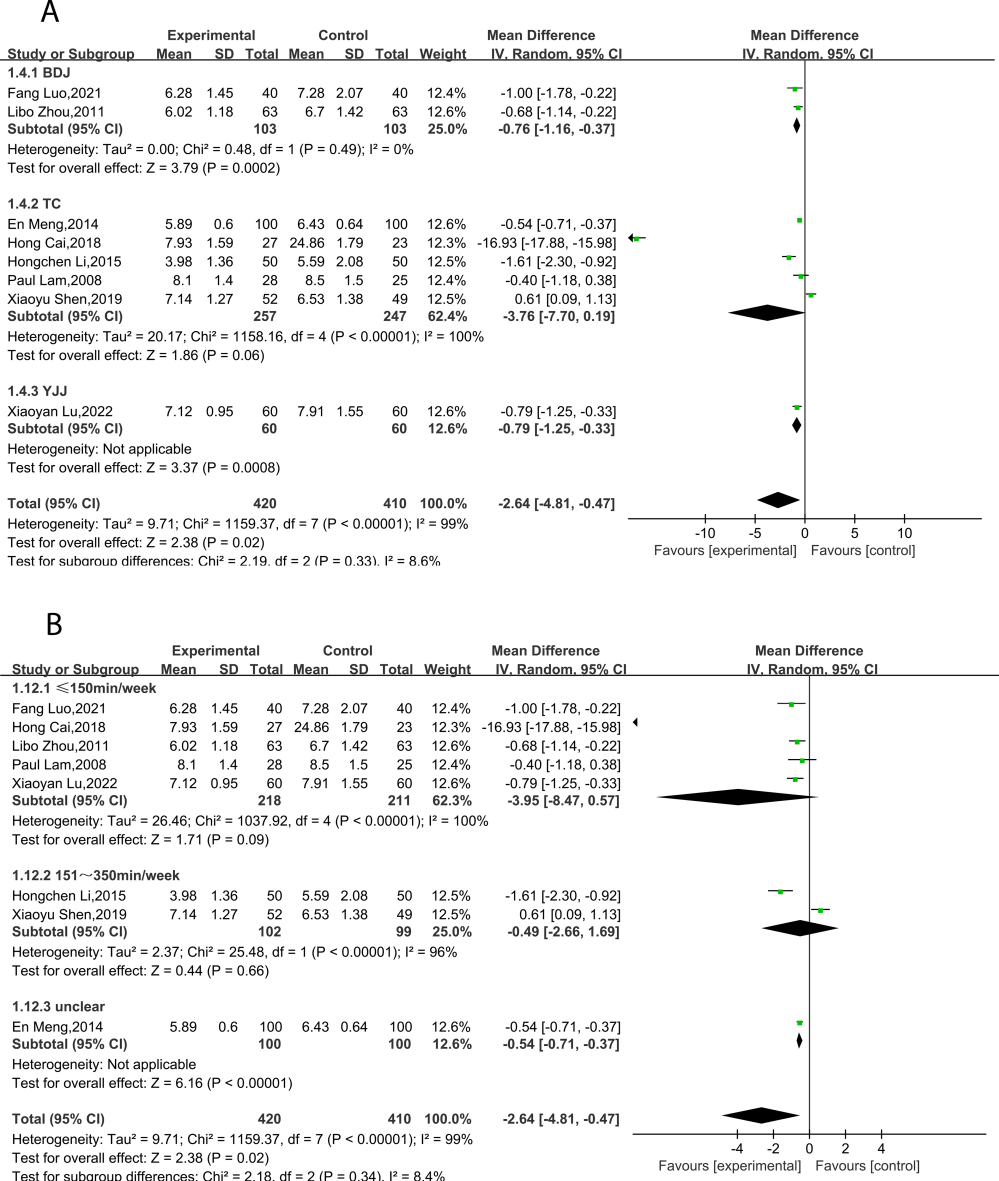
Forest plot of meta-analysis of HbA1c effect size. **(A)** Effects of different types of TCEs on HbA1c. **(B)** Effects of TCEs on HbA1c in different treatment courses.

Eight studies (23, 26–31, 33) reported HbA1c, including 830 subjects. All studies tested subjects at baseline before the trial, and the differences were not statistically significant. Heterogeneity is large (*P* < 0.00001, *I²* = 99%), therefore, a random effects model was utilized for analysis. Overall, the results demonstrated that TCEs significantly decreased HbA1c (MD = -2.64, 95% CI [-4.81, -0.47], *P* = 0.02; **Table 2**, **Figure 6**). Subgroup analyses indicated that the effect of BDJ was significantly more pronounced compared to other types of TCEs (MD = -0.76, 95% CI [-1.16, -0.37], *P* = 0.0002; **Table 2**, **Figure 7A**). Subgroup analysis based on intervention duration showed no statistically significant difference (**Table 2**, **Figure 7B**).

### 3.5 Effects of TCEs on secondary outcomes

Four studies (23, 25–27) reported 2hPG/PPG, which included 466 participants. All the study tested subjects at baseline before the trial, and the differences were not statistically significant. The combined results highlighted that TCEs considerably reduced 2hPG/PPG (MD = -1.19, 95% CI [-1.89, -0.49], *P* = 0.0008; **Table 2**). Subgroup analysis of intervention duration indicated that the greatest intervention effect could be achieved when TCEs ≤150 min/week (MD = -0.94, 95% CI [-1.82, -0.06], *P* = 0.04; **Table 2**). Five studies (23–25, 29, 31) reported BMI, including 352 participants for meta-analysis. The combined results indicated that TCEs significantly reduced BMI in individuals with DM (MD = -0.83, 95% CI [-1.42, -0.24], *P* = 0.006; **Table 2**). Subgroup analysis based on intervention duration indicated that the most significant effect of TCEs was observed with a duration of ≤150 min/week (MD = -0.76, 95% CI [-1.42, -0.11], *P* = 0.02; **Table 2**). Three studies (26, 32, 33) reported SF-36 with 291 participants in meta-analysis. The combined results demonstrated that TCEs notably improved the overall health status of older patients with DM (MD = 4.95, 95% CI [1.03, 8.87], *P* = 0.01; **Table 2**). One study (30) reported on DSQoL and the results showed that TCEs significantly improved the related quality of life of older patients with DM (MD = -5.60, 95% CI [-9.84, -1.36], *P* = 0.010; **Table 2**).

### 3.6 Adverse events

A lack of data pertaining to adverse events was observed in the studies incorporated in this review.

### 3.7 Publication bias

**Figure 8:**
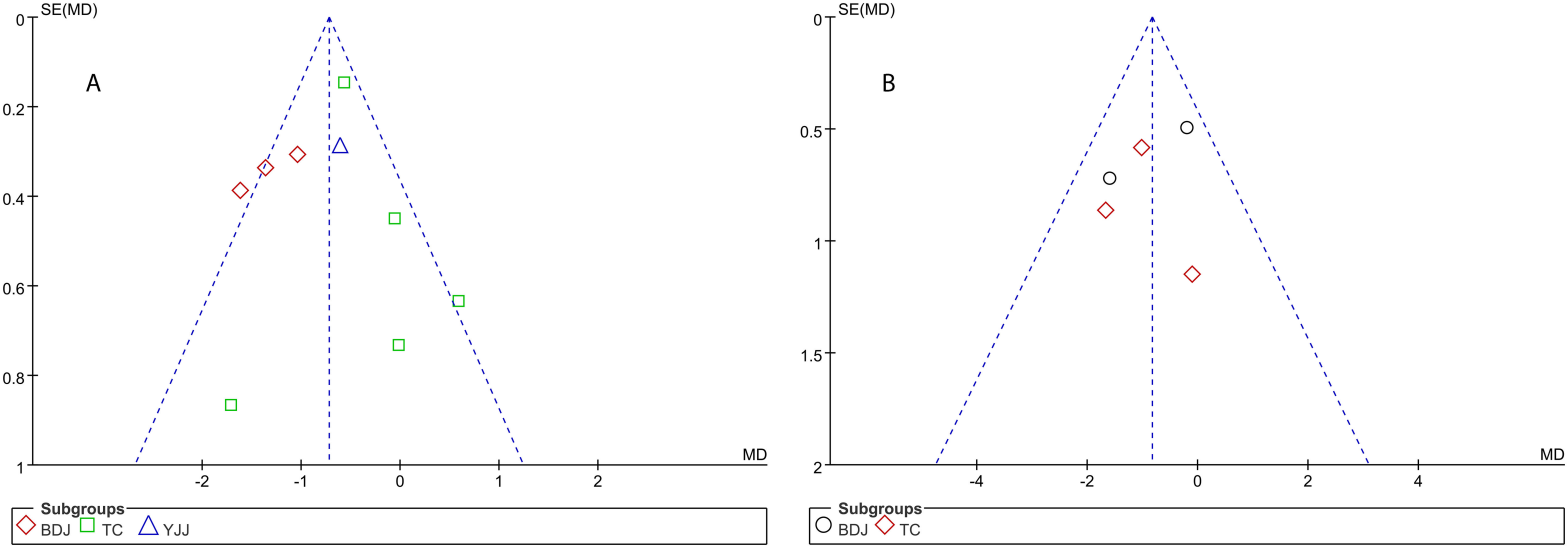
Funnel plots of effects of TCEs on FPG/FBG **(A)** and BMI **(B)** in older patients with diabetes mellitus.

The funnel plot of the effect of TCEs on FPG/FBG and BMI in older DM patients is shown in **Figure 8**. The scattered points were largely symmetric, indicating no significant publication bias.

## 4 Discussion

This research is the first systematic review and meta-analysis of the combined effects of the three most common TCEs on clinical biomarkers and health-related quality of life in older individuals with DM. The study found that TCEs reduced FPG/FBG, HbA1c, 2hPG/PPG, and BMI in older DM patients compared with controls. In addition, TCEs had a positive effect in improving health-related quality of life in older individuals with DM. Our results show that BDJ has the best overall effect compared to the other two traditional exercise therapies, especially in terms of improving FPG/FBG and HbA1c levels.

Nevertheless, the included studies exhibited high heterogeneity regarding the effects of TCEs on FPG/FBG, HbA1c, and 2hPG/PPG. The findings obtained from the meta-analysis of this study align largely with previous meta-analyses concerning the effects of single TCEs on blood glucose control in individuals with DM (34–40). For example, it has been shown that the BDJ (35) intervention positively affects FPG/FBG, HbA1c, and 2hPG/PPG in individuals with DM. However, there is no consensus on the impact of TC on blood glucose control in DM patients, with some reports suggesting that TC is more effective than other aerobic exercises in lowering HbA1c (36). The findings of the meta-analysis indicated a positive impact of TC and conventional exercise on both FPG/FBG and HbA1c in older DM patients. Furthermore, no difference was noted between the two methods. Additionally, to achieve the most effective blood glucose control through TCEs in older patients with DM, an initial investigation into the duration of the intervention was conducted by examining relevant studies. Within the studies included, the duration of TCEs varied between 12 and 24 weeks, while the frequency ranged from 2 to 7 sessions per week. Each session lasted between 30 and 60 min. The findings of this meta-analysis indicate that exercise sessions of less than 150 min/week may be the optimal duration of exercise for interventions with TCEs, which is consistent with guidelines from the American College of Sports Medicine and the American Diabetes Association (41). However, it is important to note that the small sample size and variations among the included studies may contribute to heterogeneity when combining the findings in a meta-analysis. Although the exact mechanism of inconsistent results is unclear, it may be related to factors such as intervention duration, frequency, outcome assessment, and quality control.

TCEs, as a distinct non-drug therapy in traditional Chinese medicine, encompass a form of low-intensity aerobic exercise. TCEs provide a different therapeutic mindset and approach from Western medicine. With a holistic view of the human body, TCEs prioritize harmonization as a central principle and function as physical therapy. In addition to this, TCEs have a level of participant compliance that is not present in traditional aerobic exercise (42, 43). Studies have shown that participants who perform TCEs exercises have higher completion and better compliance (44). While different forms of physical activity, including resistance exercise training, comprehensive exercise training, and high-intensity interval exercise (HIIE) (10, 45), have been shown to improve health and glycemic management in patients with type 2 diabetes mellitus (T2DM), long-term adherence to exercise programs can be challenging for older people with DM. This difficulty can arise from the high intensity of exercises and a lack of interest in participating in such programs. In contrast, TCEs exhibit diversity, ease of learning, and are low-cost, providing an active form of aerobic exercise for the older DM population.

The mechanisms regarding the impact of TCEs on blood glucose in patients with DM are multifaceted and could be attributed to several interrelated factors. TCEs, such as BDJ, TC, and YJJ, emphasize slow, controlled movements, which are inherently different from conventional exercise modalities. These exercises are rich in movement techniques and prompt regular muscle contractions and faster blood flow in DM patients. These rhythmic movements may help regulate blood glucose levels more effectively by suppressing sympathetic activation and enhancing parasympathetic activity. In addition, continuous exercise can improve the antioxidant capacity of the body (46) while reducing the level of oxidative stress. In the long run, by maintaining a certain level of exercise habits through TCEs can enhance the body’s antioxidant capacity, reduce the accumulation of reactive oxygen species (ROS), which trigger inflammatory responses in diabetic patients. Reducing oxidative stress may play a key role in improving blood glucose regulation. Furthermore, recent studies suggest that when energy arousal induced by exercise is highly activated, energy arousal dominates (47). This dynamic can be interpreted as when TCEs are practiced with a focus on self-awareness, where individuals consciously guide their own movements and thoughts, they have shown the potential to improve peripheral nerve conduction velocity in diabetic patients (48), which is particularly beneficial for diabetic patients with peripheral neuropathy. In addition to the aforementioned physiological mechanisms, the positive impact of TCEs on cognitive function has received increasing attention. Previous studies have indicated that practicing TCEs can enhance cognitive abilities in individuals with cognitive impairment to some extent. As a mind-body form of exercise, TCEs may help strike a balance between cognitive activation and psychological relaxation, thereby optimizing their overall effect on both cognitive and metabolic health. Earlier research also supports this perspective, emphasizing that interventions involving both physical and mental engagement—such as TCEs—not only facilitate cognitive activation but also enhance neural regulation, which aligns with current theoretical frameworks on cognitive enhancement interventions. On the other hand, traditional models of glucose regulation commonly suggest that even a slight drop in glucose levels may lead to reduced willpower and impaired cognitive performance (49). This decline typically occurs after high-calorie or high-carbohydrate meals, although its underlying mechanisms remain unclear. Some studies have proposed that the phenomenon may also have a habitual component. Therefore, integrating TCEs during such periods may help improve energy levels, support neural regulation, and enhance overall cognitive performance.

DM is an expanding global public health problem, especially in developing countries. The rate of diabetes control among people aged ≥65 years in China is statistically only 41.33% (50). To control DM, it is crucial to implement additional measures. To tackle the issues posed by an aging global population, the World Health Organization advocates for establishing age-friendly communities (51, 52). This approach aims to foster the growth of active aging community initiatives. community support and healthcare services are primary elements of age-friendly communities (53). To enhance or sustain the physical functional capacity of the older, redefining the healthcare approach specifically tailored for this population is essential. Thus, TCEs have great potential for community dissemination efforts in the care and prevention of older DM patients. Notably, as the global population of the older afflicted with DM continues to expand, this study obtained 7183 potentially eligible studies through the database and manual searches. However, based on the inclusion and exclusion criteria, only 11 studies encompassing 944 older patients with DM were deemed eligible for this meta-analysis. Globally, there may be a lack of scholarly attention to the health management of exercise in older adults and a lack of more in-depth mechanisms of the effects of exercise in TCEs on older adults. The standard treatment for controlling blood glucose in older diabetic patients includes oral hypoglycemic drugs and supplemental exogenous insulin. To avoid poor blood glucose control, medical staff should implement appropriate supplementary therapies and explore more suitable exercise methods for the older. Enhancing our understanding of these domains will hold great significance for the health management of older DM patients. Overall, this review may offer valuable exercise therapy options and robust evidence-based medicine for the clinical management of DM in older adults. Furthermore, it may provide fresh perspectives into the adjunctive treatment of DM with exercise therapy.

### 4.1 Limitation

However, the risk of bias in the included studies is unclear. Most included studies lacked sufficient information regarding allocation concealment, blinding, and potential follow-up bias arising from the randomized sequence. This challenge is not unique to this review but reflects a common issue in non-experimental studies, where establishing causal relationships is inherently difficult. First, the quality control of this study is unsatisfactory, which usually causes selection bias in the design and execution of clinical trials, and can reduce the credibility of meta-analyses, as treatment effects may be exaggerated by unclear allocation or failure to conduct trials in a blinded manner. Second, the sample size of the included studies was small, and most of these studies did not follow CONSORT (21) when estimating the required sample size. This may result in an increased likelihood of type II error. In addition, the results may not be generalizable as most of the participants were from the Chinese population. This study conducted a relevant literature search, and most of the interventions consisted of BDJ and TC. Few studies have examined the effects of traditional exercises such as WQX, QG, and YJJ. TCEs are an exercise modality with Chinese characteristics, and an extensive body of research has confirmed the therapeutic effects of TCEs. Therefore, it is crucial to undertake additional, comprehensive investigations on diverse modalities of TCEs to delve deeper into the underlying alterations within the internal mechanisms.

### 4.2 Outlook to research and health policy

As exercise continues to demonstrate its physiological benefits, future research should broaden its focus to explore the diverse benefits of exercise beyond blood sugar control, such as improving flexibility, cognitive function, and social interaction, particularly for aging populations. Expanding research on traditional practices like Tai Chi could uncover deeper insights into the mechanisms underlying these effects. From a health policy perspective, integrating exercise into preventive healthcare strategies could reduce chronic disease risks and healthcare costs. Policymakers should prioritize funding for research on accessible, low-cost exercise interventions, ensuring they are incorporated into public health programs to promote healthy aging and improve quality of life.

## 5 Conclusion

As the population of older people with DM continues to increase, it is critical to identify non-pharmacological treatments that can enhance the management of DM and promote overall health. TCEs have a positive impact on blood glucose control, BMI, and health-related quality of life among older people with DM. In summary, Baduanjin has significant advantages in reducing FPG/FBG and HbA1c. TCEs was most effective in improving FPG/FBG levels when exercise time was less than 150 minutes per week. Therefore, in health management, appropriate TCEs can be selected for exercise according to different types and needs of patients. Overall, our findings add to the evidence supporting the use of TCEs to reduce measures of glycemic control.
