# Supplementary material for: Effect of traditional Chinese exercise on older patients with diabetes mellitus: a systematic review and meta-analysis of randomized controlled trials
Source: Front Endocrinol (Lausanne). 2025 May 8;16:1499051. doi: 10.3389/fendo.2025.1499051 (PMC12094918; doi:10.3389/fendo.2025.1499051)
Supplement: Supplementary file 1 [file DataSheet1.docx]

Supplementary Material

**Search strategy**

**1. Search strategy for PubMed:**

#1 Search "Diabetes Mellitus"[Mesh]

#2 Search "Diabetes Mellitus, Type 2"[Mesh]

#3 Search: ((((((((((((((((((((((((((((((Diabetes Mellitus, Noninsulin-Dependent[Title/Abstract]) OR (Diabetes Mellitus, Ketosis-Resistant[Title/Abstract])) OR (Diabetes Mellitus, Ketosis Resistant[Title/Abstract])) OR (Ketosis-Resistant Diabetes Mellitus[Title/Abstract])) OR (Diabetes Mellitus, Non Insulin Dependent[Title/Abstract])) OR (Diabetes Mellitus, Non-Insulin-Dependent[Title/Abstract])) OR (Non-Insulin-Dependent Diabetes Mellitus[Title/Abstract])) OR (Diabetes Mellitus, Stable[Title/Abstract])) OR (Stable Diabetes Mellitus[Title/Abstract])) OR (Diabetes Mellitus, Type II[Title/Abstract])) OR (NIDDM[Title/Abstract])) OR (Diabetes Mellitus, Noninsulin Dependent[Title/Abstract])) OR (Diabetes Mellitus, Maturity-Onset[Title/Abstract])) OR (Diabetes Mellitus, Maturity Onset[Title/Abstract])) OR (Maturity-Onset Diabetes Mellitus[Title/Abstract])) OR (Maturity Onset Diabetes Mellitus[Title/Abstract])) OR (MODY[Title/Abstract])) OR (Diabetes Mellitus, Slow-Onset[Title/Abstract])) OR (Diabetes Mellitus, Slow Onset[Title/Abstract])) OR (Slow-Onset Diabetes Mellitus[Title/Abstract])) OR (Type 2 Diabetes Mellitus[Title/Abstract])) OR (Noninsulin-Dependent Diabetes Mellitus[Title/Abstract])) OR (Noninsulin Dependent Diabetes Mellitus[Title/Abstract])) OR (Maturity-Onset Diabetes[Title/Abstract])) OR (Diabetes, Maturity-Onset[Title/Abstract])) OR (Maturity Onset Diabetes[Title/Abstract])) OR (Type 2 Diabetes[Title/Abstract])) OR (Diabetes, Type 2[Title/Abstract])) OR (Diabetes Mellitus, Adult-Onset[Title/Abstract])) OR (Adult-Onset Diabetes Mellitus[Title/Abstract])) OR (Diabetes Mellitus, Adult Onset[Title/Abstract])

#4 Search "Diabetes Mellitus, Type 1"[Mesh]

#5 Search (((((((((((((((((((Diabetes Mellitus, Insulin-Dependent[Title/Abstract]) OR (Diabetes Mellitus, Insulin Dependent[Title/Abstract])) OR (Insulin-Dependent Diabetes Mellitus[Title/Abstract])) OR (IDDM[Title/Abstract])) OR (Diabetes Mellitus, Sudden-Onset[Title/Abstract])) OR (Diabetes Mellitus, Sudden Onset[Title/Abstract])) OR (Sudden-Onset Diabetes Mellitus[Title/Abstract])) OR (Type 1 Diabetes Mellitus[Title/Abstract])) OR (Diabetes Mellitus, Insulin-Dependent, 1[Title/Abstract])) OR (Insulin-Dependent Diabetes Mellitus 1[Title/Abstract])) OR (Insulin Dependent Diabetes Mellitus 1[Title/Abstract])) OR (Type 1 Diabetes[Title/Abstract])) OR (Diabetes, Type 1[Title/Abstract])) OR (Diabetes Mellitus, Type I[Title/Abstract])) OR (Autoimmune Diabetes[Title/Abstract])) OR (Diabetes Mellitus, Brittle[Title/Abstract])) OR (Brittle Diabetes Mellitus[Title/Abstract])) OR (Diabetes Mellitus, Ketosis-Prone[Title/Abstract])) OR (Ketosis-Prone Diabetes Mellitus[Title/Abstract])) OR (Diabetes Mellitus, Ketosis Prone[Title/Abstract])

#6 #1 OR #2 OR #3 OR #4 OR #5

#7 Search (((((((((((((((((((((((((((((((Mind Body Therapies[Title/Abstract]) OR (Mind-Body Therapy[Title/Abstract])) OR (Therapies, Mind-Body[Title/Abstract])) OR (Therapy, Mind-Body[Title/Abstract])) OR (Mind-Body Medicine[Title/Abstract])) OR (Mind Body Medicine[Title/Abstract])) OR (traditional exercise[Title/Abstract])) OR (traditional chinese medicine[Title/Abstract])) OR (chinese traditional exercise[Title/Abstract])) OR (traditional chinese exercise[Title/Abstract])) OR (chinese exercise[Title/Abstract])) OR ("Tai Ji"[Mesh])) OR (Tai-ji[Title/Abstract])) OR (Tai Chi[Title/Abstract])) OR (Chi, Tai[Title/Abstract])) OR (Tai Ji Quan[Title/Abstract])) OR (Ji Quan, Tai[Title/Abstract])) OR (Quan, Tai Ji[Title/Abstract])) OR (Taiji[Title/Abstract])) OR (Taijiquan[Title/Abstract])) OR (T'ai Chi[Title/Abstract])) OR (Tai Chi Chuan[Title/Abstract])) ) OR ("Qigong"[Mesh])) ) OR (Qi Gong[Title/Abstract])) OR (Ch'i Kung[Title/Abstract])) OR (baduanjin[Title/Abstract])) OR (wuqinxi[Title/Abstract])) OR (yijinjing[Title/Abstract])

#8 #6 AND #7 984

**2. Search strategy for Web of Science**

1: TS=( "Diabetes Mellitus")

2: TS=("Diabetes Mellitus, Type 2"OR"Diabetes Mellitus, Noninsulin-Dependent" OR "Diabetes Mellitus, Ketosis-Resistant"OR "Diabetes Mellitus, Ketosis Resistant" OR "Ketosis-Resistant Diabetes Mellitus"OR "Diabetes Mellitus, Non Insulin Dependent"OR "Diabetes Mellitus, Non-Insulin-Dependent"OR "Non-Insulin-Dependent Diabetes Mellitus"OR "Diabetes Mellitus, Stable" OR "Stable Diabetes Mellitus"OR "Diabetes Mellitus, Type II" OR "NIDDM"OR "Diabetes Mellitus, Noninsulin Dependent"OR "Diabetes Mellitus, Maturity-Onset"OR "Diabetes Mellitus, Maturity Onset"OR "Maturity-Onset Diabetes Mellitus" OR "MODY"OR "Diabetes Mellitus, Slow-Onse"OR "Diabetes Mellitus, Slow Onset"OR "Slow-Onset Diabetes Mellitus"OR "Type 2 Diabetes Mellitus"OR "Noninsulin-Dependent Diabetes Mellitus"OR "Noninsulin Dependent Diabetes Mellitus"OR "Maturity-Onset Diabetes"OR "Diabetes, Maturity-Onset"OR "Maturity Onset Diabetes"OR "Type 2 Diabetes"OR "Diabetes, Type 2" OR "Diabetes Mellitus, Adult-Onset"OR "Adult-Onset Diabetes Mellitus"OR "Diabetes Mellitus, Adult Onset")

3: TS=("Diabetes Mellitus, Type 1"OR"Diabetes Mellitus, Insulin-Dependent"OR "Diabetes Mellitus, Insulin Dependent"OR "Insulin-Dependent Diabetes Mellitus"OR "IDDM" OR "Diabetes Mellitus, Sudden-Onset" OR "Diabetes Mellitus, Sudden Onset" OR "Sudden-Onset Diabetes Mellitus"OR "Type 1 Diabetes Mellitus"OR "Diabetes Mellitus, Insulin-Dependent, 1" OR "Insulin-Dependent Diabetes Mellitus 1"OR "Insulin Dependent Diabetes Mellitus 1"OR "Type 1 Diabetes"OR "Diabetes, Type 1"OR "Diabetes Mellitus, Type I" OR "Autoimmune Diabetes"OR "Diabetes Mellitus, Brittle"OR "Brittle Diabetes Mellitus" OR "Diabetes Mellitus, Ketosis-Prone" OR "Ketosis-Prone Diabetes Mellitus" OR "Diabetes Mellitus, Ketosis Prone")

4: #1 OR #2 OR #3

5: TS=("Mind Body Therapies"OR "Mind-Body Therapy" OR "Therapies, Mind-Body"OR "Therapy, Mind-Body" OR "Mind-Body Medicine" OR "Mind Body Medicine"OR "traditional exercise" OR "traditional chinese medicine"OR "chinese traditional exercise" OR "traditional chinese exercise"OR "chinese exercise"OR "Tai Ji"OR "Tai-ji"OR "Tai Chi"OR "Chi, Tai" OR "Tai Ji Quan" OR "Ji Quan, Tai"OR "Quan, Tai Ji"OR "Taiji"OR "Taijiquan" OR "Tai Chi Chuan" OR "Qigong"OR "Qi Gong" OR "Ch'i Kung"OR "baduanjin"OR "wuqinxi" OR "yijinjing")

6: #4 AND #5 2047

1. **Search strategy for Wanfang Data Knowledge Service Platform**

#1 题名或关键词:("糖尿病" or "高血糖" or "血糖高 " or "1型糖尿病" or "2型糖尿病")

#2 题名或关键词:("太极拳" or "太极" or "八段锦 " or "五禽戏" or "易筋经" or "气功" or "传统运动" or "中国传统运动" or "中医传统功法" or "中医功法" or "中国传统运动疗法" or "身心运动")

#3 #1 and #2 360

**4. Search strategy for China National Knowledge Infrastructure**

#1 AB = 糖尿病 OR AB = 高血糖 OR AB = 血糖高 OR AB = 1型糖尿病 OR AB = 2型糖尿病

#2 SU=太极拳 OR SU= 太极 OR SU=八段锦 OR SU=五禽戏 OR SU=易筋经 OR SU=气功 OR SU=传统运动 OR SU=中国传统运动 OR SU=中医传统功法 OR SU=中医功法 OR SU=中国传统运动疗法 OR SU=身心运动

#3 #1 AND #2 569

**5.Search strategy for Cochrane Library**

#1 MeSH descriptor: [Diabetes Mellitus] explode all trees

#2 MeSH descriptor: [Diabetes Mellitus, Type 2] explode all trees

#3 Type 2 Diabetes:ti,ab,kw OR Type 2 Diabetes Mellitus:ti,ab,kw OR Maturity Onset Diabetes Mellitus :ti,ab,kw OR Diabetes Mellitus, Maturity-Onset:ti,ab,kw OR Diabetes Mellitus, Type II:ti,ab,kw OR Diabetes, Maturity-Onset :ti,ab,kw OR Diabetes Mellitus, Slow-Onset :ti,ab,kw OR Non-Insulin-Dependent Diabetes Mellitus :ti,ab,kw OR Noninsulin Dependent Diabetes Mellitus :ti,ab,kw OR Maturity-Onset Diabetes Mellitus :ti,ab,kw OR Diabetes Mellitus, Maturity Onset :ti,ab,kw OR Ketosis-Resistant Diabetes Mellitus :ti,ab,kw OR Diabetes Mellitus, Noninsulin Dependent:ti,ab,kw OR Diabetes Mellitus, Adult-Onset:ti,ab,kw OR NIDDM:ti,ab,kw OR Diabetes Mellitus, Non Insulin Dependent :ti,ab,kw OR Adult-Onset Diabetes Mellitus :ti,ab,kw OR Slow-Onset Diabetes Mellitus:ti,ab,kw OR Diabetes Mellitus :ti,ab,kw OR Noninsulin-Dependent :ti,ab,kw OR Diabetes, Type 2 :ti,ab,kw OR MODY :ti,ab,kw OR Stable Diabetes Mellitus :ti,ab,kw OR Diabetes Mellitus, Ketosis-Resistant :ti,ab,kw OR Maturity-Onset Diabetes :ti,ab,kw OR Diabetes Mellitus, Adult Onset:ti,ab,kw OR Noninsulin-Dependent Diabetes Mellitus :ti,ab,kw OR Maturity Onset Diabetes :ti,ab,kw OR Diabetes Mellitus, Slow Onset :ti,ab,kw OR Diabetes Mellitus, Ketosis Resistant :ti,ab,kw OR Diabetes Mellitus, Non-Insulin-Dependent :ti,ab,kw OR Diabetes Mellitus, Stable:ti,ab,kw

#4 MeSH descriptor: [Diabetes Mellitus, Type 1] explode all trees

#5 Ketosis-Prone Diabetes Mellitus :ti,ab,kw OR Diabetes Mellitus, Ketosis-Prone :ti,ab,kw OR Diabetes Mellitus, Ketosis Prone:ti,ab,kw OR Diabetes Mellitus, Insulin-Dependent :ti,ab,kw OR Diabetes Mellitus, Juvenile-Onset :ti,ab,kw OR Diabetes Mellitus, Sudden-Onset :ti,ab,kw OR Juvenile-Onset Diabetes Mellitus :ti,ab,kw OR Sudden-Onset Diabetes Mellitus :ti,ab,kw OR Insulin-Dependent Diabetes Mellitus 1:ti,ab,kw OR IDDM :ti,ab,kw OR Diabetes Mellitus, Insulin Dependent :ti,ab,kw OR Insulin Dependent Diabetes Mellitus 1:ti,ab,kw OR Diabetes Mellitus, Sudden Onset :ti,ab,kw OR Type 1 Diabetes Mellitus :ti,ab,kw OR Diabetes, Type 1 :ti,ab,kw OR Type 1 Diabetes :ti,ab,kw OR Insulin-Dependent Diabetes Mellitus :ti,ab,kw OR Diabetes Mellitus, Insulin-Dependent, 1 :ti,ab,kw OR Diabetes Mellitus, Type I :ti,ab,kw OR Diabetes Mellitus, Brittle:ti,ab,kw OR Brittle Diabetes Mellitus :ti,ab,kw OR Diabetes, Autoimmune :ti,ab,kw OR Autoimmune Diabetes :ti,ab,kw

#6 #1 OR #2 OR #3 OR #4 OR #5

#7 MeSH descriptor: [Tai Ji] explode all trees

#8 Tai Chi :ti,ab,kw OR T'ai Chi :ti,ab,kw OR Taiji :ti,ab,kw OR Tai-ji:ti,ab,kw OR Taijiquan:ti,ab,kw OR Chi, Tai :ti,ab,kw OR Quan, Tai Ji :ti,ab,kw OR Tai Ji Quan :ti,ab,kw OR Tai Chi Chuan:ti,ab,kw OR Ji Quan, Tai:ti,ab,kw

#9 MeSH descriptor: [Qigong] explode all trees

#10 Ch'i Kung :ti,ab,kw OR Qi Gong:ti,ab,kw

#11 baduanjin :ti,ab,kw OR wuqinxi :ti,ab,kw OR yijinjing :ti,ab,kw OR Mind Body Therapies :ti,ab,kw OR Mind-Body Therapy:ti,ab,kw OR Therapies, Mind-Body:ti,ab,kw OR Therapy, Mind-Body :ti,ab,kw OR Mind-Body Medicine:ti,ab,kw OR Mind Body Medicine :ti,ab,kw OR traditional exercise :ti,ab,kw OR traditional chinese medicine :ti,ab,kw OR chinese traditional exercise :ti,ab,kw OR traditional chinese exercise :ti,ab,kw OR chinese exercise:ti,ab,kw

#12 #7 OR #8 OR #9 OR #10 OR #11

#13 #6 and #12 1226

1. **Search strategy for Embase**

#1 'diabetes mellitus'/exp

#2 'non insulin dependent diabetes mellitus'/exp

#3 ('adult onset diabetes':ab,ti OR 'adult onset diabetes mellitus':ab,ti OR 'diabetes mellitus type 2':ab,ti OR 'diabetes mellitus type ii':ab,ti OR 'diabetes mellitus, maturity onset':ab,ti OR 'diabetes mellitus, non insulin dependent':ab,ti OR 'diabetes mellitus, non-insulin-dependent':ab,ti OR 'diabetes mellitus, type 2':ab,ti OR 'diabetes mellitus, type ii':ab,ti OR 'diabetes type 2':ab,ti OR 'diabetes type ii':ab,ti OR 'diabetes, adult onset':ab,ti OR 'dm 2':ab,ti OR 'insulin independent diabetes':ab,ti OR 'insulin independent diabetes mellitus':ab,ti OR 'ketosis resistant diabetes mellitus':ab,ti OR 'maturity onset diabetes':ab,ti OR 'maturity onset diabetes mellitus':ab,ti OR 'maturity onset diabetes of the young':ab,ti OR 'niddm':ab,ti OR niddm:ab,ti) AND 'non insulin dependent diabetes mellitus':ab,ti OR 'non insulin dependent diabetes':ab,ti OR 'non-insulin-dependent diabetes mellitus':ab,ti OR 'noninsulin dependent diabetes':ab,ti OR 'noninsulin dependent diabetes mellitus':ab,ti OR 't2dm':ab,ti OR 'type 2 diabetes':ab,ti OR 'type 2 diabetes mellitus':ab,ti OR 'type ii diabetes':ab,ti OR 'type ii diabetes mellitus':ab,ti

#4 'insulin dependent diabetes mellitus'/exp

#5 'brittle diabetes':ab,ti OR 'brittle diabetes mellitus':ab,ti OR 'diabetes mellitus type 1':ab,ti OR 'diabetes mellitus type i':ab,ti OR 'diabetes mellitus, brittle':ab,ti OR 'diabetes mellitus, insulin dependent':ab,ti OR 'diabetes mellitus, insulin-dependent':ab,ti OR 'diabetes mellitus, juvenile onset':ab,ti OR 'diabetes mellitus, type 1':ab,ti OR 'diabetes mellitus, type i':ab,ti OR 'diabetes type 1':ab,ti OR 'diabetes type i':ab,ti OR 'diabetes, juvenile':ab,ti OR 'dm 1':ab,ti OR 'early onset diabetes mellitus':ab,ti OR 'iddm':ab,ti OR 'insulin dependent diabetes':ab,ti OR 'insulin-dependent diabetes mellitus':ab,ti OR 'juvenile diabetes':ab,ti OR 'juvenile diabetes mellitus':ab,ti OR 'juvenile onset diabetes':ab,ti OR 'juvenile onset diabetes mellitus':ab,ti OR 'ketoacidotic diabetes':ab,ti OR 'labile diabetes mellitus':ab,ti OR 'mckusick 22210':ab,ti OR 't1dm':ab,ti OR 'type 1 diabetes':ab,ti OR 'type 1 diabetes mellitus':ab,ti OR 'type i diabetes':ab,ti OR 'type i diabetes mellitus':ab,ti

#6 #1 OR #2 OR #3 OR #4 OR #5

#7 'tai chi'/exp

#8 'tai chi chuan':ab,ti OR 'tai ji':ab,ti OR 'taiji quan':ab,ti OR 'taijiquan':ab,ti

#9 'qigong'/exp

#10 'chi kung':ab,ti OR 'chigung':ab,ti OR 'qi gong':ab,ti

#11 'baduanjin':ti,ab OR 'wuqinxi':ti,ab OR 'yijinjing':ti,ab OR 'traditional exercise':ti,ab OR 'traditional chinese medicine':ti,ab OR 'chinese traditional exercise':ti,ab OR 'traditional chinese exercise':ti,ab OR 'chinese exercise':ti,ab

#12 'mind body medicine'/exp

#13 #7 OR #8 OR #9 OR #10 OR #11 OR #12

#14 #6 AND #13 1995
